# Improving efficiency in hospital pharmacy systems: the case for color-coded zones for rapidly dispensed medications

**DOI:** 10.1080/20523211.2025.2493169

**Published:** 2025-04-24

**Authors:** Ammar Abdulrahman Jairoun, Sabaa Saleh Al-Hemyari, Moyad Shahwan, Faris El-Dahiyat, Abeer M. Al-Ghananeem

**Affiliations:** a Health and Safety Department, Dubai Municipality, Dubai, UAE; b Discipline of Clinical Pharmacy, School of Pharmaceutical Sciences, Universiti Sains Malaysia (USM), Pulau Pinang, Malaysia; c Pharmacy Department, Emirates Health Services, Dubai, UAE; d Department of Clinical Sciences, College of Pharmacy and Health Sciences, Ajman University, Ajman, United Arab Emirates; e Centre of Medical and Bio-allied Health Sciences Research, Ajman University, Ajman, United Arab Emirates; f Clinical Pharmacy Program, College of Pharmacy, Al Ain University, Al Ain, United Arab Emirates; g AAU Health and Biomedical Research Center, Al Ain University, Abu Dhabi, United Arab Emirates

**Keywords:** Hospital pharmacy, colour-coded zones, emergency medications, patient safety

## Abstract

Colour-coded zones for rapidly dispensed medications are a simple, yet game-changing solution to improve the efficiency and safety of hospital pharmacy systems. This approach integrates visual ergonomics, staff training, and technology in solving some of the most common challenges in medication retrieval and general healthcare delivery. This evidence-based system will go a long way in helping hospitals meet the ever-increasing demands. While colour-coded zoning schemes offer a practical and cost-effective way to enhance pharmacy efficiency and medication safety, their effectiveness depends on careful implementation, standardisation, and integration with digital tools. Although highly digitalised healthcare systems may rely more on automated inventory tracking and barcode verification, colour coding remains a valuable supplementary measure, particularly in fast-paced clinical environments. For resource-limited healthcare facilities, colour-coded zoning serves as an essential safety mechanism where advanced IT solutions may not be feasible. However, standardisation challenges, risks of over-reliance, and accessibility concerns for colour-blind individuals must be addressed to maximise effectiveness. Ultimately, an optimal pharmacy management system may involve a hybrid approach that integrates both visual and digital verification methods, ensuring both efficiency and patient safety across diverse healthcare settings.

## Background

In the dynamic environment of hospital pharmacies, efficient drug dispensing is critical to patient safety and operational effectiveness (Institute for Safe Medication Practices, 2018; Nizame et al., 2021). Incompetence in this process can cause delays, increased workloads, and a higher risk of medication errors and miscalculations (Keers et al., 2013). Antibiotics, analgesics, and emergency pharmaceuticals are among the most frequently dispensed medications (Nizame et al., 2021; Pedersen et al., 2017). Medications needing rapid dispensing typically include emergency drugs, critical care medications, or drugs needed in life-threatening situations.

The pharmaceutical zones in pharmacies and hospitals are medications kept according to their type, function, or urgency. This makes it easier for medical personnel to find and obtain the appropriate medications when they're needed. Establishing a colour-coded zoning system is a novel, evidence-based approach to improving workflow efficiency, reducing retrieval time, and increasing safety (International Health Facility Guidelines, 2023; Tecsys, 2025). Research has shown that colour coding can reduce errors in high-stress environments by leveraging the brain’s ability to recognise and react to colours more swiftly than text, with each colour corresponding to a specific type or urgency level of medication (Kohn et al., 2000).

This editorial looks at the theoretical foundations, practical applications, and broader implications of this method. Furthermore, it will serve as a cornerstone for further scientific investigations and guiding the direction for policy recommendations.

## Concept and principles of colour-coded zones

Colour-coded zones rely on cognitive psychology and visual ergonomics, where the role of colour plays an important factor in enhancing visual cues by reducing cognitive overload (Ware, 2021; Wickens & Hollands, 2000). Designating unique colours to the type of medication or medication retrieval area assists the pharmacy staff in improving the visual order, increasing access speed, and reducing the incidence of errors (Thomas & Tee, 2022). Colours act as instantaneous identifiers for locating medications even for busy pharmacy assistants (Bradford Systems, n.d.; Stanton et al., 2019). This organisation method minimises the chances of selecting the wrong medication, thus increasing accuracy and safety (Grasha & Schell, 2001; Holden et al., 2021).

## System design and implementation

The initial process for a colour-coded zone organisation includes analysing the workflow of the pharmaceuticals within the pharmacy. There must be an audit performed that identifies the quickly dispensed medications based on prescription frequency and criticality and classifies them as high, medium, or low-demand classes (McDonough & Doucette, 2003; Pedersen et al., 2017). After the audit has been completed, a designated specific area of the pharmacy or medication storage system must be colour-coded. For example, high-demand emergency medications may be assigned to a red zone, medium-demand drugs to a yellow zone, and low-demand products to a blue zone (Bradford Systems, n.d.; Holden et al., 2021). Highly visible signs and labels are essential in guiding the navigation of staff (Stanton et al., 2019; Wickens & Hollands, 2000). Following the design of the zoning system, personnel training and consistency are critical to successful implementation.

Pharmacy staff must grasp the rationale behind the colour-coded system and receive training to integrate it into their daily workflows (Lane et al., 2006; Murray et al., 2019). Standard operating procedures (SOPs) should be updated to reflect the new system, and ongoing training programmes can maximise its benefits (Institute for Safe Medication Practices, 2021; Kohn et al., 2000). Additionally, integrating the colour-coded zone system into the Electronic Health Record (EHR) system and Automated Dispensing Units (ADUs) can further streamline operations. Barcode scanning in conjunction with the zoning system increases verification and tracking (Al-Worafi, 2023; Bates & Gawande, 2003).

## Practical applications and advantages

Colour-coded zones have been highly effective in various settings, both in the hospital pharmacy and ambulatory care environments (Bradford Systems, n.d.). During codes, where every minute counts, staff can quickly locate lifesaving medications such as epinephrine or thrombolytics because of designated areas, thus minimising delays in treatment (Grasha & Schell, 2001; Lane et al., 2006). Clinics with a high turnover of patients will benefit from the speed of dispensing frequently prescribed drugs such as insulin and antihypertensives (Pedersen et al., 2017). The system enhances inventory management by visually monitoring understocked areas, thereby facilitating restocking and reducing the incidence of expired medications in stock (Institute for Safe Medication Practices, 2021; Wickens & Hollands, 2000). Evidence exists to support the effectiveness of colour-coded zoning systems. One study, as reported in *The American Journal of Health-System Pharmacy*, demonstrated that within six months of operation, retrieval times were reduced by 35%, along with a 20% reduction in dispensing errors (Bradford Systems, n.d.; Holden et al., 2021). Staff questionnaires reflected increased confidence and greater satisfaction with the management of medications, further supporting the system's operational benefits (Stanton et al., 2019).

## Global relevance and implementation across healthcare systems

The applicability of colour-coded zoning systems varies across countries due to differences in regulatory frameworks, healthcare infrastructure, and cultural attitudes toward safety measures.

In highly digitalised healthcare systems such as those in the United States, Canada, Australia, New Zealand, and much of Western Europe, the widespread use of automated dispensing cabinets and electronic medication administration records has reduced the need for visual zoning. However, even in these advanced settings, colour-coded systems remain a valuable secondary safety measure, particularly in high-demand hospital areas such as emergency departments and anesthetic clinics, where rapid medication identification is critical (Gebremariam et al., 2023).

For instance, anesthesiologists in the United States, Canada, the United Kingdom, and Australia have adopted colour-coding for user-applied syringe labels in accordance with the International Anaesthetic Labelling Standard, introduced in 2008 to reduce medication errors through standardised label colours, sizes, and designs (Filiatrault & Hyland, 2009; Noorily & Overdyk, 20%). Despite this, debate persists regarding the true effectiveness of colour-coded systems, as studies have produced mixed results on their impact in reducing errors.

In contrast, low- and middle-income countries that continue to rely on manual or semi-automated pharmacy management systems often find colour-coded zoning a cost-effective and practical method for streamlining drug distribution and minimising retrieval errors. For these healthcare settings, where implementing advanced digital inventory systems may not be financially feasible, visual zoning serves as a simple yet effective safety mechanism.

A one-size-fits-all approach is impractical due to variations in healthcare systems, regulatory requirements, and patient safety protocols. Additionally, cultural perceptions of colour may influence implementation strategies, requiring flexible adaptation to local needs. Further research is necessary to examine the impact of different colour schemes on workflow efficiency and medication safety across diverse healthcare environments.

To ensure that colour-coded zoning serves as a complementary, rather than standalone, approach in modern pharmacy practice, future studies should focus on comparing its efficacy across various healthcare settings. Such research will be instrumental in refining global adoption strategies and optimising medication management systems worldwide.

## Challenges and strategies for risk management

Despite its benefits, implementing a colour-coded system may encounter challenges such as upfront costs, staff resistance, and spatial constraints. It is important to note that implementation necessitates investment in signage, training, and technical enhancement (Bradford Systems, n.d.; Lane et al., 2006). It is necessary to develop clear procedures that explain what each colour represents and how to navigate the storage and dispensing areas quickly.

A phased rollout starting with high-risk medications can help achieve quick successes and build confidence and support for the system. It is far easier to fix issues early on and expand gradually to ensure long-term success (McDonough & Doucette, 2003). Gradually rolling out the system with high-risk drugs is a logical approach due to the significant risk of harm in the event of an error. High-risk medications, such as insulin, opioids, and anticoagulants, pose a greater threat to patient safety if used improperly (WA Health, 2025). Implementing a colour-coded zoning system for these pharmaceuticals allows healthcare facilities to prioritise areas requiring the most safety enhancements. This strategy minimises errors while ensuring that staff can quickly and accurately access these medications under pressure (Graham et al., 2008). Focusing first on high-risk drugs also enables early detection of systemic issues, providing opportunities for improvement before expanding to other drug classes. Evidence from similar programmes suggests that prioritising high-risk areas leads to measurable reductions in medication errors and increased staff confidence, fostering broader system adoption (McDonough & Doucette, 2003).

Staff engagement, early and ongoing education, allows overcoming resistance to move away from workflow (Murray et al., 2019; Wickens & Hollands, 2000). Compact shelving systems for space-limited facilities and reserving space for highly demanded medicines can enhance the effectiveness of the arrangement with less spatial re-arrangement (Pedersen et al., 2017; Stanton et al., 2019). Monitoring performance is highly recommended through regular audits or surveys so that changes to the zone system can be updated accordingly.

## Alternatives and comparisons with IT-based solutions

The evolution of hospital pharmacy management has been driven by advanced IT-based systems that enhance the tracking, retrieval, and storage of medications (Cheng et al., 2023). Automated inventory systems, barcode scanning, and RFID tracking technologies enable real-time monitoring of drug supplies, reducing human error and improving efficiency. From the perspective of the Healthcare Information and Management Systems Society (HIMSS), fully digitalised inventory systems may seem like the optimal solution.

However, while these technologies offer numerous advantages, quick and intuitive visual identification remains essential. In high-stress environments such as emergency rooms and intensive care units, pharmacy staff often find that immediate visual cues are more efficient than navigating digital interfaces or scanning barcodes (Brown et al., 2019). Additionally, colour-coded zoning presents a cost-effective and practical alternative, particularly for hospitals and smaller healthcare facilities with limited resources that cannot afford or implement complex IT infrastructures.

Rather than viewing digital inventory systems and colour-coded zoning as competing approaches, an integrated system that combines both may offer the best balance of efficiency and accuracy.

That said, the potential drawbacks of colour-coding schemes must be carefully assessed. False reassurance is a primary concern, as medical personnel may rely solely on colour identification rather than verifying medication labels, increasing the risk of errors, particularly under pressure (Merry et al., 2020). Furthermore, colour vision impairments could lead to misinterpretations, making accessibility an important consideration (Merry et al., 2020).

Another significant challenge is the lack of standardisation – inconsistent colour scheme application across manufacturers or hospital departments can create confusion, increasing the risk of misidentification (Nemec, 2009). Alternative technology solutions, such as barcode scanning for syringe labels, provide a more reliable method for medication verification (Merry et al., 2020). Additionally, real-time tracking technologies and modern inventory management systems offer robust solutions for improving efficiency and medication safety.

In summary, while colour-coded zoning can enhance workflow efficiency and provide an additional layer of safety, it should be complemented by digital verification tools to create a more comprehensive and error-resistant approach to hospital pharmacy management.

## Implications for broader pharmaceutical practice

The impacts of colour-coded zones exceed operation efficiency. They immediately promote patient safety and clinical outcomes by facilitating the timely availability of vital medications (Al-Worafi, 2023). The pharmacy can manage increased patient volumes with better quality of services. Implementing colour-coded zones in pharmacies immediately enhances patient safety, operational efficiency, and therapeutic outcomes, raising the standard of care. These systems improve medication accessibility, reducing treatment delays and minimising the risk of pharmaceutical misidentification (Al-Worafi, 2023). Research also indicates that colour-coded systems enhance task performance by enabling faster drug identification and retrieval, especially in high-pressure situations (APS Foundation, 2025; WHA, 2007). This improvement in workflow efficiency allows pharmacies to manage higher patient volumes while maintaining high standards of care (Pedersen et al., 2017).

The approach is scalable, suitable for more extensive facilities, and adaptable for specialised departments such as pediatrics or oncology, where distinctive pharmaceutical needs arise (McDonough & Doucette, 2003; Stanton et al., 2019). Besides that, the system also can best conform to regulatory standards set by organisations such as The Joint Commission, International Health Facility Guidelines (iHFG), U.S. Food and Drug Administration's Code of Federal Regulations (FDA CFR) Part 201, and the International Organization for Standardization (ISO) 15223-1 (Bates & Gawande, 2003; Institute for Safe Medication Practices, 2021). While the benefits of implementing colour-coded zones in hospital pharmacy systems – such as improved efficiency and safety – are evident, it is essential to recognise potential challenges and limitations. A major concern is the cost and logistical effort required to redesign storage spaces and train staff in the new system. Hospitals already using computerised inventory management systems may view the transition as unnecessary, particularly if their existing systems efficiently facilitate medication retrieval.

Additionally, colour-coded zoning may pose accessibility challenges for individuals with colour vision impairments, necessitating supplementary labelling or alternative identification methods. Consistent staff adherence and ongoing maintenance are also critical; without proper reinforcement, the system’s effectiveness may decline over time.

Regulatory and patient safety organisations, including the American Medical Association (AMA), Food and Drug Administration (FDA), Institute for Safe Medication Practices (ISMP), and American Society for Health-System Pharmacists (ASHP), have expressed concerns regarding the widespread adoption of colour-coding practices (Merry et al., 2020). One major obstacle is the difficulty of maintaining uniform colour schemes, particularly as an increasing number of prefilled and prelabeled syringes from different manufacturers introduce inconsistencies. The limited number of commercially available distinguishable colours further complicates matters, as more nuanced differentiation may be required (Merry et al., 2020).

Moreover, some patient safety organisations argue that there is insufficient evidence to conclusively prove that colour coding reduces medication errors. Reports of misinterpretations underscore the potential risks of implementing such systems without complementary safeguards (Filiatrault & Hyland, 2009). These considerations highlight the importance of a structured and controlled approach when integrating colour-coded zoning into pharmacy operations, ensuring that it serves as an adjunct to, rather than a replacement for, other safety measures.

## Conclusion

While colour-coded zoning schemes offer a practical and cost-effective way to enhance pharmacy efficiency and medication safety, their effectiveness depends on careful implementation, standardisation, and integration with digital tools. Although highly digitalised healthcare systems may rely more on automated inventory tracking and barcode verification, colour coding remains a valuable supplementary measure, particularly in fast-paced clinical environments.

For resource-limited healthcare facilities, colour-coded zoning serves as an essential safety mechanism where advanced IT solutions may not be feasible. However, standardisation challenges, risks of over-reliance, and accessibility concerns for colour-blind individuals must be addressed to maximise effectiveness.

Ultimately, an optimal pharmacy management system may involve a hybrid approach that integrates both visual and digital verification methods, ensuring both efficiency and patient safety across diverse healthcare settings.

## Call to action

As a recommendation, this is a call for the implementation of a colour-coded system while ensuring all stakeholders are involved, guidelines and standards are followed, technology is integrated, and continuous improvement is encouraged. Pharmacy leads from different institutions should test a colour-coded zoning system and provide feedback to build a strong evidence-based system. After that, the system should be refined to become a global standard for how modern pharmacies should operate.
